# Factors Associated with the Health Literacy on Social Determinants of Health: A Focus on Socioeconomic Position and Work Environment

**DOI:** 10.3390/ijerph17186663

**Published:** 2020-09-13

**Authors:** Mikyeong Cho, Young-Me Lee, Seung joo Lim, Hyeonkyeong Lee

**Affiliations:** 1 College of Nursing, Yonsei University, 50-1, Yonsei-ro, Seodaemun-gu, Seoul 03722, Korea; zzomi324@naver.com; 2 School of Nursing, DePaul University, Chicago, IL 60640, USA; YLEE23@depaul.edu; 3 Department of Nursing, Research Institute for Basic Science, Hoseo University, 20 Hoseo-ro, 79 beon-gil, Asan 31499, Korea; seungju62@hanmail.net

**Keywords:** social determinants of health, health literacy, socioeconomics factors, occupational health nursing

## Abstract

Though impressive progress has been made in healthcare worldwide, many people still experience disproportionate health burdens and inequities in healthcare services. For establishing sustainable development goals, health literacy on social determinants of health (HL-SDH) has been recognized as a key to creating better social and physical environments. In particular, low levels of health literacy among industrial workers are considered as a major barrier to benefitting from the progress made in healthcare. This study aimed to describe levels of HL-SDH and to examine the relationships among socioeconomic status, working environment, and HL-SDH among workers in Korea. A total of 660 workers from an online panel participated in Korea from 30 May to 7 June 2018. The Korean version of HL-SDH instrument consisted of 33 items and four dimensions: access, understand, appraise, and apply. Descriptive statistics, a t-test, ANOVA, and multiple regression analysis were conducted. The mean score of HL-SDH was 2.48. Perceived mental health status, annual income, and the number of employees per worksite were found to be statistically significant factors related to HL-SDH (F = 3.64, *p* = <0.001). The HL-SDH score was considerably higher, indicating that nursing interventions to enhance HL-SDH are required for health promotion, especially among workers in poor social and working environments.

## 1. Introduction

Health disparities between different populations are known to be influenced by individual biological characteristics such as individual genetic characteristics and health-associated lifestyles. Aside from these factors, various other factors such as regional, environmental, and social characteristics in complex interaction play a great role in determining health status [1]. In the Sustainable Development Goals (SDGs) for 2030 that were presented to the 2015 United Nations (UN) General Assembly, health status is regarded as a performance index of sustainable development in terms of economic, social, and environmental aspects. In particular, through the achievement of SDGs, awareness of the social determinants of health (SDH) is gaining importance in the macroscopic perspective to prevent infectious and non-communicable diseases, and to reduce health inequalities. As the World Health Organization (WHO) Shanghai Declaration [2], is an essential component in the achievement of the SDGs for 2030, so SDH warrants attention from health-related experts and researchers.

SDH refers to the “wider set of forces and systems comprising of such states as a human being born, growing, working, living, and shaping the conditions of daily life” [3]. SDH is regarded as the ultimate cause because it combines interactions of various causes via social mechanisms [4]. For example, residing in an impoverished area increases a person’s exposure to climatic environmental factors such as air pollution as well as inadequate food sources. There are also cases in which access to healthcare services is low because of the increased exposure time to workplace hazards due to prolonged working hours [4]. Thus, SDH should not be disregarded in workers’ healthcare because health risks can be increased by increased exposure to combinations of multiple other factors.

The WHO’s Commission on Social Determinants of Heath (CSDH) presents SDH in a conceptual framework, identifies triggering factors for health disparities, and explains how the main factors are interrelated [1]. The core components in this conceptual framework are structural and interventional factors. The structural factors are associated with socioeconomic and sociopolitical contexts and socioeconomic status. These affect interventional factors such as the physical environment, health behavior, sociopsychological factors, and public health, leading to health disparities through interactions. Health disparities affect structural and interventional factors. Social integration and social capital play intermediary roles between structural and interventional factors [5]. Such SDHs are distributed unequally according to education, occupation, income, social class, gender, race, and ethnicity, which have important places in research and policymaking as the main concepts for achieving health goals in terms of health equity reconsideration [4].

Health literacy refers to the “ability to understand the basic health information and to receive and manage health services that allow an individual to make appropriate healthcare decisions.” [6]. According to a systemic review of 96 studies, low health literacy was associated with reduced use of healthcare services and low health performance [7]. In these studies, health literacy is considered as an interventional factor of SDH and the approach so far to treating health literacy has been a major concept in previous literature.

The effect of health literacy on social determinants of heath (HL-SDH) is “not only limited to health promotion related to SDH, but means a combination of abilities to access, understand, appraise, and apply” [8]. This concept integrates health literacy and health determinants, referring to the ability to access, understand, appraise, and apply health information in terms of health promotion [9]. It is a more expanded concept of health literacy, but differs from health literacy in that it is not solely about the use of health information to determine the general health status of individuals, instead, it includes the ability to seek, understand, assess, and apply information in a community which can affect health. This includes factors such as drinking water, housing, transportation, and climate. Reducing health inequities caused by health disparities requires the acquisition of health information capabilities regarding SDH [8]. Thus, HL-SDH includes measurable interventional factors such as physical environment, health behavior, sociopsychological factors in conceptual framework [1].

Workers in production age groups represent a larger group than population groups in the other lifecycle stages. Therefore, they are more likely to be exposed to SDH such as socioeconomic status and working environment that can affect their health in various ways. Income is the representative index that plays a definitive role in identifying socioeconomic status. The most urgent priority for low-income-status people is making a living, ahead of health management, so these people usually report low health status [10,11]. Moreover, though low-income individuals may try to lead healthy lifestyles, they typically lack the capacity to purchase nutritious foods or the time for exercise compared to upper-class individuals [12].

The work environment, including the worker’s job category, employment type, workplace size, and workplace health management support system, is also important for both individuals’ health issues and in terms of the industrial nursing aspect of the community. Thus, resolving health issues and health protection for workers through access to SDH are critical. A recent study emphasized the importance of SDH for worker’s health because workers who comprise a large portion of the adult population assume various roles in the family, at work, and in the community [13]. Although they are the population group most heavily affected by SDH in various ways, research into SDH for workers has been limited recently. Therefore, research into HL-SDH, that is, workers’ awareness of SDH and related characteristics, is necessary

This study aims to describe levels of HL-SDH and to examine the relationships among socioeconomic status, working environment, and HL-SDH among workers in Korea.

## 2. Materials and Methods

This is a descriptive study that describes levels of HL-SDH, and examines the relationships among socioeconomic status, working environment, and HL-SDH among workers in Korea.

### 2.1. Participants

The subjects in this study were adult workers aged 20–64 years, who were recruited from a nationwide online panel carried out by a specialized survey research agency from 30 May to 7 June 2018. Subjects were native Korean speakers who had no difficulty speaking, reading, or writing, understood the study purpose, and participated in the study voluntarily via an online survey. Because 24.8% of older adults in Korea lack basic reading and writing abilities, which is a large difference from younger and middle-aged adults in terms of basic information literacy [14], adults in this study 65 years old and older were excluded from data collection due to their presumed difficulty in adequately discerning the data.

Subject screening questions comprised four questions that included employment status, gender (male/female), region (metropolitan area/non-metropolitan area), and age, whereas an equal number of participants for each group of gender, region, and age were sampled. A total of 660 participants were recruited through online research panels. There were no missing values such as “no response”, so we used the data obtained from all 660 participants for the final data analysis. The method has been described in more detail elsewhere [15] and the entire procedure was approved by the IRB (No. Y-2017-3357-004). Online informed consent was received from all subjects prior to their survey participation.

### 2.2. HL-SDH

The HL-SDH instrument was developed by Matsumoto and Nakayama [8], and we used the original instrument with the author’s permission. An original instrument was translated into Korean, comprising 33 questions in four subdomains, i.e., seven questions for “access”, seven questions for “understanding”, seven questions for “appraisal”, and 12 questions for “application.” Each answer was a four-point Likert scale (1 = very difficult, 2 = difficult, 3 = easy, and 4 = very easy). We added a “not know/not applicable” item so that we could assess each participant’s level as in the original instrument. The selection of this item was treated as a missing value, as in the original instrument, and these values were replaced by the average value for all responders. We used a weighted average by adding all the scores for each question and dividing this by the number of questions. When arranging the selection view in each question—as in the original instrument—HL-SDH was lower for higher scores, so we performed reverse coding, as in the original instrument. For both the instrument at the time of development and the translated instrument in Korean, the Cronbach-α was 0.92. The Korean version of HL-SDH (K-HL-SDH)’s reliability was appropriate and acceptable [15].

### 2.3. Socioeconomic Status

The representative index for identifying socioeconomic status refers to social class, gender, ethnicity, education, occupation, and income [5]. This study utilized a four-item questionnaire about gender, education level, occupation, and income. The questions asking about occupation covered these categories: (1) specialty professional (e.g., professor, physician, or lawyer), (2) government official, (3) teacher/lecturer, (4) service/sales (e.g., airlines worker, hotel worker, call center worker, banker, or beautician), (5) operation/management (e.g., corporate executive or higher), (6) office worker (e.g., corporate director or below), (7) production/skills/labor (e.g., repair worker, driver, or engineer), and (8) freelancer (e.g., artist). The occupation was re-categorized by a type of job for analysis as “office worker” for specialty professional, government official, teacher/lecturer, operation/management, and office worker, “production” for service/sales and production/skills/labor, and “freelancer” for freelancer.

For social class, we referred to the criteria used in Kim et al. [12] and stratified according to occupation and employment type. In this study, upper- or upper–middle-class (class I) corresponds to “specialty professional and operation/management,” which includes senior executives, specialized professionals, business owners, employers, and regular employees. The new middle-class (class II) corresponds to “government official, teacher/lecturer, office worker, and production/skills/labor,” which includes mental laborers, office workers, employers, and daily employees. The old middle-class (class III) is made up of the self-employed among service or sales workers, skills workers, and machine operators. In this study, there was no separate survey question to confirm self-employed status, so we regarded “freelancers” as self-employed for categorization. The labor class (class IV) corresponded to “service/sales,” which includes regular, temporary, or daily workers in service or sales. For our study, there were no self-employed people among the agriculture/fishery workers who corresponded to class V, nor were there simple laborers or employees among the agriculture/fishery workers who correspond to class VI.

Education level was recategorized into below high school and high school and above. The average annual income was based on minimum wage in 2018, 20 million KRW as a reference, and recategorized into <20 million KRW, 20–<30 million KRW, 30–<40 million KRW, and ≥40 million KRW.

### 2.4. Work Environment

Work environment was one of the interventional factors in the conceptual framework of SDH [5], so it is necessary to investigate the characteristics of work environments. This study used eight questions for work environment: employment period, job title, regular employment (y/n), full-time employment (y/n), shift work (y/n), weekend shift (y/n), and workplace size. Workplace size refers to the number of people.

### 2.5. General Characteristics

Seven questions were used to inquire into workers’ general characteristics: age, marital status, living with a spouse (y/n), religion, and region. Perceived physical health status and perceived mental health status were each one question. Each answer was a four-point Likert scale.

### 2.6. Data Analysis

Collected data were treated using SPSS/WIN 23.0. The frequency and percentage were calculated to assess HL-SDH in terms of the participants’ general characteristics, socioeconomic status, and work environment. A *t*-test or analysis of variance was performed to distinguish the level of HL-SDH based on the subject’s general characteristics, socioeconomic status, and work environment; a Bonferroni test was performed for post-verification.

To investigate correlations between workers’ socioeconomic status, work environment, and HL-SDH level, multiple regression analysis was performed by using the enter method with subjective health status, income level, and workplace size as independent variables, which showed significant differences in univariate analysis, with HL-SDH as the dependent variable.

## 3. Results

### 3.1. Participant’s General Characteristics

Married workers were 65.9% of all the participants, and those living together with family were 79.5%. Participants who were religious accounted for 49.5%, and metropolitan and non-metropolitan subjects were sampled at an equal rate of 50%, respectively. Regarding perceived physical health status, 57.7% answered that it was good. Regarding perceived mental health status, 72.4% answered that it was good, comprising a large portion, and they displayed significantly higher HL-SDH than the others (t = 2.24, *p* = 0.026) (Table 1).

### 3.2. Level of Workers’ HL-SDH

Workers’ HL-SDH was a minimum of 1.15 and maximum of 3.82, and the mean score was 2.48 ± 0.42. Among the subfactors, the mean score of “understanding” was the highest at 2.83 ± 0.61, followed by “access” at 2.53 ± 0.51, “appraisal” at 2.49 ± 0.54, and “application” at 2.27 ± 0.51.

In analyzing each question, out of all 33 questions, the mean score of item 12, “Understanding job insecurity can become highly stressful,” was the highest at 3.05 ± 0.86, followed by item 9, “Understanding the fact that childhood abuse can affect you in your adulthood,” at 2.99 ± 0.85. Both questions were in the “understanding” category. On the other hand, the mean score of item 27, “Asking politicians or administrative institutions for the reformation of rules and regulations for workers’ health protection,” was 2.06 ± 0.83, and item 31, “Asking politicians or administrative institutions for easier treatments for illegal drug users,” was 2.07 ± 0.78, both of which were in the “apply” category and scored the lowest (Table 2).

### 3.3. Disparities in HL-SDH of Heath Based on Workers’ Socioeconomic Status

The social class of most participants was new middle class (class II) at 64.7%, with 50% of each sex due to the equal sampling. As for education level, those who had completed high school and above made up 98.8%.

For job-related characteristics, office work was the most common at 64.7%, and those who made less than 20 million KRW annually, the minimum wage according to the 2018 standard, made up 18.3%. Workers who made 30–<40 million KRW a year, compared with those who made 40 million KRW and over, showed significantly higher HL-SDH (t = 3.58, *p* = 0.014) (Table 3).

### 3.4. Disparities in the HL-SDH Based on Work Environment

Those with work experience of less than 10 years accounted for 53.2%. Staff employees were 42.3%, permanent employees were 69.7%, and full-time employees were 82.4%, which was the largest portion. Shift workers were 13.5%, weekend-only workers were 30.8%, and overtime workers (more than 40 h a week on average) were 65.0%. A small workplace of fewer than 50 employees accounted for 62.4%, comprising the highest proportion, and HL-SDH was significantly higher for workplaces with 100–300 employees than for those with fewer than 50 employees (t = 2.68, *p* = 0.046) (Table 3).

### 3.5. Factors Affecting the HL-SDH

To confirm the factors influencing HL-SDH, the independent variables were set as perceived mental health status, income, and workplace size because they showed significant differences in the univariate analysis of HL-SDH. Additionally, to control the income difference between full-time and hourly work, hourly work was inserted as a variable and HL-SDH was set as an independent variable. When confirming the basic hypothesis of regression analysis to judge multicollinearity, the Durbin–Watson statistic was 2.09, meeting the mutual independence of residuals requirement. The tolerance level was 0.63–0.98, which was greater than 0.1, and the range of the variance inflation factor (VIF) was 1.03–1.60, which was less than 10; hence, there was no issue with multicollinearity. Multiple regression analysis in the form of the batch input method was performed, and a total of four variables were inserted in the analysis. As a result, the variables that exerted a significant influence on HL-SDH were perceived mental health status (β = −0.08, *p* = 0,044), an annual income of 30 to 40 million KRW (β = 0.16, *p* = <0.001), and a workplace size from 100 to 300 employees (β = 0.12, *p* = 0.002). The F value of the regression model was 3.64 (*p* = <0.001), so the regression model was adequate, and the modified decision coefficient (adjusted R^2^) was 3%. Thus, HL-SDH was shown to be lower when perceived mental health status was higher in all categories. The HL-SDH was higher for the 30 to 40 million KRW income group than for those earning less than 30 million KRW and more than 40 million KRW, HL-SDH was shown to be lower for workplaces with fewer than 50 people than those with 100 to 300 people (Table 4).

## 4. Discussion

Workers spend more of their time at workplaces than other population groups, so the resolution of health-related problems and health protection at the workplace are of importance, but research about the HL-SDH is scarce. This study first presents the influence of SDH, which is of great significance for HL-SDH to analyze the degrees of HL-SDH that workers recognize and related characteristics.

In this study, the degree of HL-SDH for Korean workers was higher than half of the total score, but there were some discrepancies depending on the item. The score for “understanding” was the highest, followed by “access”, “appraisal”, and “application”. These are the same results as those from the study in Japan that showed a high “understanding” but low “application” where the instrument was originally developed [8]. Through these results, we can learn that regarding understanding and accessing SDH, it is relatively easy for workers to recognize and become aware of their ability to carry this out, but they find it difficult to assess and apply information.

A direct comparison of the total scores and scores by area of HL-SDH between the two studies in two different countries is difficult. An investigation of the methods suggested by each study provided the following insights. We found that most of the items of the lowest mean score in our study matched the “difficult” and “very difficult” items in Japan, which were the “application” items. Thus, we can see that the subjects found it difficult to apply SDH-related information. In particular, subjects talked to their supervisors or employers regarding the most difficult job efforts and their inadequate salary. This reflects the Korean organizational culture in the workplace, where the workers do not feel that they can make requests or express their opinions freely to their employer. Moreover, there were many cases in which workers found it difficult to make requests directed at politicians or administrative institutions about improving rules and regulations for health protection. Workers have a greater interest in income for living, including work environment. They may find it difficult to do because they tend to avoid requests directed at politicians or administrative institutions unless their health deteriorates and they receive a diagnosis of occupational disease, or they are approved for an industrial injury. Many participants felt that it was difficult to make a request directed at politicians or administrative institutions regarding easier access to treatments for illegal drug users in the future. It seems that there are few opportunities and limited accessibility because it is difficult to meet illegal drug users in their daily lives or at a workplace within Korea and the social issue regarding illegal drugs is relatively new, unlike in some other countries [16]. Moreover, someone who is socially isolated and damaging their own health is hard to find, which could be why workers’ perceive access as difficult in daily life. Understanding that the expansion of income differences between workers may damage people’s relationships is high, but participating in poverty removal activities is difficult for them.

On the other hand, we found that most of the “understanding” items had the highest mean score per question, as in the items “easy” and “very easy” in the Japanese case. Considering that the score for understanding that job insecurity is a large source of stress was the highest, Korean workers may be aware that unreasonable things at work are stressful to them. Nevertheless, they tolerate the stress and do not act easily because their stress over job insecurity is greater. Additionally, the score for understanding that childhood abuse can exert an influence in adulthood was high. We think that there was an awareness and understanding about the importance of childhood environment since all participants were adults and some were married with children. Understanding that increasing income difference damages people’s relationships matched the results in this study about the significant relationship between income and HL-SDH.

Subjective health status is used as an important index predictive of health level and is the variable that measures physical and mental health statuses combined. According to the original instrument study [8], the higher the subjective health status, the higher the HL-SDH. This study was conducted by dividing the factors into subjective physical health status and subjective mental health status: the lower the subjective mental health status, the higher the HL-SDH. Moreover, subjective mental status represents the psychosocial factor, which is an interventional factor within the SDH conceptual framework. Therefore, it should be considered important when a health intervention is carried out after the subject’s characteristics are recognized.

In this study, HL-SDH was low among workers who reported low income levels. This is because income is the representative index and a factor deciding socioeconomic status, and the lower the income level, the harder it is to pay attention to health. Previous studies have reported that the lower the income, the lower the health literacy [10,11], whereas in this study, the HL-SDH was higher for those earning 30 to 40 million KRW than for those earning less than 30 million KRW and more than 40 million KRW. There are many previous studies on the relationship between income and health literacy, but there is a lack of research related to HL-SDH. In the study using the original instrument, income and HL-SDH were found to have no significant correlation, so future research needs to continuously connect income to it.

In this study, HL-SDH was shown to be lower in workplaces with fewer than 50 people than in those with 100 to 300. This means that workplace size can have an influence. Workplaces with fewer than 50 employees, comprising most workplaces in South Korea, show high rates of industrial disasters, placing them in the blind zone of health management. In South Korea, small workplaces of fewer than 50 workers are not required to appoint a health manager and at best they receive health screenings or regular visits for health counseling from external health agencies. Additionally, in many cases, there is an absence of a systematic and regular health promotion program. Therefore, it is important to establish a health management system for the workers in a small workplace with a worker health center under national supervision, and to investigate how to expand this program.

As mentioned above, the health level is affected by not only individual biological and lifestyle aspects but also the social structure and related factors. Nonetheless, there has been a scarcity of studies relating to SDH in worker’s health regarding who is most influenced by various social structures and factors. In this study, we conducted research using the Korean-translated HL-SDH instrument for the first time in the country. The K-HL-SDH instrument may have been somewhat difficult to understand because it contains some questions that the study participants may not have considered in daily life. Additionally, there is a need for continuous refinement of the instrument through various population in local communities in the future.

## 5. Conclusions

This study is the first conducted in Korea that utilized a K-HL-SDH instrument. In this study, in particular, it was shown that HL-SDH is affected by factors such as average annual income and workplace size. Workers in a workplace of 100 to 300 employees showed significantly higher HL-SDH than those in a small workplace of 50 people or fewer. For average annual income, workers who received 30 to 40 million KRW annually showed significantly higher HL-SDH than those receiving 40 million KRW. Based on the above results, it is confirmed that income, as well as workers’ access to the work environment, is needed to improve Korean workers’ HL-SDH. Based on the results of this study, we suggest a direction for future research and nursing practices to assess and improve the HL-SDH of Korean workers. First, this study is limited because the participants were Korean workers who were selected through an online survey research agency. Thus, the study results should not be generalized to all workers. We therefore suggest research investigating various phenomena for different degrees of HL-SDH for various types of workers. Second, this study concerned various workers as subjects, so groups of certain occupations may have different subject characteristics. Thus, we suggest a future study in which workers’ job categories are classified to assess the degrees of SDH factors and confirm their effects. Lastly, this study confirmed that income, a socioeconomic index for workers, and workplace size, a work environment index, were important factors affecting SDH, so we suggest that nursing intervention strategies should be implemented that reflect the socioeconomic status and work environment characteristics to improve workers’ health in the community.

## Figures and Tables

**Table 1:** Difference in HL-SDH by general characteristics (*N* = 660).

Variables	Characteristics	Categories	N (%)	HL-SDH	
				M ± SD	t or F (*p*)
General character-ristics	Age (year)	20–29	132 (20.0)	2.54 ± 0.47	1.42 (0.227)
		30–39	132 (20.0)	2.42 ± 0.37	
		40–49	132 (20.0)	2.47 ± 0.46	
		50–59	132 (20.0)	2.50 ± 0.40	
		60–64	132 (20.0)	2.48 ± 0.42	
	Marital status	No	225 (34.1)	2.52 ± 0.43	1.24 (0.289)
		Yes	373 (56.5)	2.46 ± 0.42	
		Divorced or Separated	62 (9.4)	2.46 ± 0.42	
	Living together or not	Alone	135 (20.5)	2.53 ± 0.44	1.43 (0.240)
		With Family	408 (61.8)	2.46 ± 0.42	
		Acquaintance	117 (17.7)	2.50 ± 0.42	
	Religion	Yes	327 (49.5)	2.50 ± 0.41	1.19 (0.234)
		No	333 (50.5)	2.46 ± 0.44	
	Region	Metropolitan	330 (50.0)	2.49 ± 0.43	0.45 (0.717)
		Chungcheong-do	49 (7.4)	2.42 ± 0.38	
		Gyeongsang-do	63 (9.6)	2.47 ± 0.42	
		Jeolla-do	218 (33.0)	2.48 ± 0.43	
	Perceived physical health status	Good	381 (57.7)	2.49 ± 0.44	0.89 (0.374)
		Poor	279 (42.3)	2.46 ± 0.41	
	Perceived mental health status	Good	478 (72.4)	2.50 ± 0.44	2.24 (0.026)
		Poor	182 (27.6)	2.42 ± 0.39	

M = mean; SD = standard deviation.

**Table 2:** Level of HL-SDH among Workers (*N* = 660).

Subscale	Item		M ± SD
**Access**	1	Find out about the impact of social position on health	2.38 ± 0.76
	2	Find information related to the impact of the daily life of a mother on the growth of the child to be born	2.70 ± 0.82
	3	Find someone who is isolated from society and whose health is failing	2.18 ± 0.81
	4	Find information on the relation between unemployment and stress	2.59 ± 0.82
	5	Find out the support required by someone in trouble in the community or workplace	2.32 ± 0.78
	6	Find out smoking is not going to eliminate the cause of stress	2.70 ± 0.85
	7	Find information about the relationship between dietary changes and health	2.87 ± 0.81
	**TTL**		**2.53 ± 0.51**
**Understand**	8	Understand that the lesser the income the greater the tendency to become ill	2.64 ± 0.83
	9	Understand that abuse suffered as a child has an impact even when one becomes an adult	2.99 ± 0.85
	10	Understand that being isolated from the community and workplace impacts health	2.78 ± 0.82
	11	Understand that determining how to proceed working on one’s own is related to stress	2.66 ± 0.81
	12	Understand that work that is not stable becomes a huge stress	3.05 ± 0.86
	13	Understand that widening income disparities dilute the ties between people	2.91 ± 0.84
	14	Understand that in a society with a high level of stress, there is a tendency toward dependency on drugs	2.79 ± 0.86
	**TTL**		**2.83 ± 0.61**
**Appraise**	15	Judge what inequities exist in society in view of living a healthy life	2.55 ± 0.82
	16	Judge what kind of government services should be supplied to those really in need of support	2.42 ± 0.81
	17	Judge what level of burden of work has on health	2.51 ± 0.87
	18	Judge what kind of support should be supplied to someone in trouble in the community or workplace	2.36 ± 0.75
	19	Judge how neighbors should help each other	2.47 ± 0.79
	20	Judge the merits and demerits of the spread of processed foods	2.48 ± 0.82
	21	Judge the kind of impact that motorization has on health	2.56 ± 0.82
	**TTL**		**2.49 ± 0.54**
**Apply**	22	Cooperate in the creation of a fair society in which everyone can live a healthy life	2.33 ± 0.84
	23	Involve oneself in politics and public administration to help small children live a healthy life	2.23 ± 0.80
	24	Participate in childcare support activities	2.32 ± 0.78
	25	Participate in activities to eliminate poverty	2.21 ± 0.82
	26	Involve oneself in politics and public administration to protect the health of workers both institutionally and legally	2.06 ± 0.83
	27	Approach the manager or the employer regarding rewards that do not match efforts done at work	1.92 ± 0.79
	28	Participate in activities to increase employment and vocational training opportunities	2.26 ± 0.75
	29	Participate in activities that support an individual, including his or her family, who is in trouble in the community or workplace	2.23 ± 0.76
	30	Participate in activities to spread the importance of ties with people for health	2.40 ± 0.76
	31	Involve oneself in politics and public administration to make it easier for persons who have used illegal drugs to receive treatment	2.07 ± 0.78
	32	Participate in activities that promote a healthy diet	2.64 ± 0.79
	33	Involve oneself in politics and public administration to seek road priority for pedestrians and cyclists	2.29 ± 0.81
	**TTL**		**2.27 ± 0.51**
**HL-SDH (Total of 33 items)**			**2.48 ± 0.42** **(** **Range 1.15** **–3.82)**

**Table 3:** HL-SDH by socioeconomic position and work environment among workers. (*N* = 660).

Variables	Characteristics	Categories	*N* (%)	HL-SDH	
				M	t or F (*p*)
Socioeconomic position	Social stratum group ^†^	Class I	92 (13.9)	2.47 ± 0.40	1.34 (0.259)
		Class II	427 (64.7)	2.50 ± 0.43	
		Class III	45 (6.8)	2.43 ± 0.45	
		Class IV	96 (14.6)	2.42 ± 0.39	
	Gender	Male	330 (50.0)	2.49 ± 0.45	0.35 (0.724)
		Female	330 (50.0)	2.47 ± 0.40	
	Education level	<High school	8 (1.2)	2.31 ± 0.46	−1.16 (0.245)
		≥High school	652 (98.8)	2.48 ± 0.42	
	Job category ^‡^	Office Job	427 (64.7)	2.49 ± 0.43	0.65 (0.523)
		Blue Collar Job	188 (28.5)	2.46 ± 0.41	
		Freelancer	45 (6.8)	2.43 ± 0.45	
	Annual income (million KRW)	<20	121 (18.3)	2.46 ± 0.41	3.58 (0.014) b < a
		20–<30	171 (25.9)	2.47 ± 0.43	
		30–<40 ^a^	135 (20.5)	2.58 ± 0.44	
		≥40 ^b^	233 (35.3)	2.44 ± 0.41	
Work environment	Years of employment	<10	351 (53.2)	2.49 ± 0.43	0.11 (0.898)
		10–<20	166 (25.1)	2.47 ± 0.45	
		≥20	143 (21.7)	2.48 ± 0.40	
	Job class	Staff	279 (42.3)	2.50 ± 0.43	1.02 (0.309)
		≥Manager	381 (57.7)	2.47 ± 0.42	
	Employment status	Permanent	460 (69.7)	2.47 ± 0.43	−0.84 (0.401)
		Temporary	200 (30.3)	2.50 ± 0.41	
	Full time or part time	Full time	544 (82.4)	2.47 ± 0.42	−1.48 (0.140)
		Part time	116 (17.6)	2.53 ± 0.44	
	Shift duty	Yes	89 (13.5)	2.51 ± 0.45	0.77 (0.443)
		No	571 (86.5)	2.48 ± 0.42	
	Work in weekends	Yes	203 (30.8)	2.47 ± 0.42	−0.26 (0.796)
		No	457 (69.2)	2.48 ± 0.43	
	Working time (week)	≤40	231 (35.0)	2.50 ± 0.39	0.67 (0.501)
		>40	429 (65.0)	2.47 ± 0.44	
	Worksite size	<50 ^c^	412 (62.4)	2.46 ± 0.42	2.68 (0.046) c < d
		50–<100	83 (12.6)	2.46 ± 0.40	
		100–<300 ^d^	71 (10.8)	2.60 ± 0.47	
		≥300	94 (14.2)	2.51 ± 0.43	

M = mean; SD = standard deviation. ^†^ Social stratum group. Class I (upper- or upper–middle-class): specialty professional and operation/management; class II (new middle-class): government official, teacher/lecturer, office worker, and production/skills/labor; class III (old middle-class): the self-employed among service or sales workers, skills workers, and machine operators; class IV (labor class): “service/sales” which includes regular, temporary, or daily workers. ^‡^ Job category. Office Job: (1) specialty professional (e.g., professor, physician, or lawyer), (2) government official, (3) teacher/lecturer, (4) operation/management (e.g., corporate executive or higher), (5) office worker (e.g., corporate director or below). Blue collar job: (1) service/sales (e.g., airline worker, hotel worker, call center worker, banker, or beautician), (2) production/skills/labor (e.g., repair worker, driver, or engineer) ·Freelancer: freelancer (e.g., artist).^a^: 30–<40, ^b^: ≥40, ^c^: <50, ^d^: 100–<300. The HL-SDH was significantly higher for the “a” income group than for those earning less than “b”; The HL-SDH was shown to be significantly lower for workplaces with fewer than “c” than those with “d”.

**Table 4:** Multiple regression for factors predicting HL-SDH (*N* = 660).

Variables	Characteristics	Categories	HL-SDH	
			β	*p*
General characteristic	Perceived mental health status		−0.08	0.044
Socioeconomic position	Annual income (million KRW) (ref: ≥40)	<20	0.04	0.461
		20–<30	0.07	0.139
		30–<40	0.16	<0.001
Work environment	Full time or part time (ref: full time)	Part time	0.08	0.063
	Worksite size (ref: <50)	50–<100	0.02	0.589
		100–<300	0.12	0.002
		≥300	0.07	0.106
Adjusted R^2^ = 0.03, F = 3.64, *p* = <0.001				

Ref = reference group.
